# Differentiation of Spontaneous Bacterial Peritonitis from Secondary Peritonitis in Patients with Liver Cirrhosis: Retrospective Multicentre Study

**DOI:** 10.3390/diagnostics13050994

**Published:** 2023-03-06

**Authors:** Silvia Würstle, Alexander Hapfelmeier, Siranush Karapetyan, Fabian Studen, Andriana Isaakidou, Tillman Schneider, Roland M. Schmid, Stefan von Delius, Felix Gundling, Rainer Burgkart, Andreas Obermeier, Ulrich Mayr, Marc Ringelhan, Sebastian Rasch, Tobias Lahmer, Fabian Geisler, Paul E. Turner, Benjamin K. Chan, Christoph D. Spinner, Jochen Schneider

**Affiliations:** 1 Department of Internal Medicine II, University Hospital Rechts der Isar, School of Medicine, Technical University of Munich, Ismaninger Str. 22, 81675 Munich, Germany; 2 Department of Ecology and Evolutionary Biology, Yale University, 165 Prospect Street, New Haven, CT 06520, USA; 3 Institute of General Practice and Health Services Research, School of Medicine, Technical University of Munich, Ismaninger Str. 22, 81675 Munich, Germany; 4 Institute of AI and Informatics in Medicine, School of Medicine, Technical University of Munich, Einsteinstr. 25, 81675 Munich, Germany; 5 Department of Internal Medicine II, RoMed Hospital Rosenheim, Pettenkoferstr. 10, 83022 Rosenheim, Germany; 6 Department of Gastroenterology, Hepatology, and Gastrointestinal Oncology, Bogenhausen Hospital of the Munich Municipal Hospital Group, Englschalkinger Straße 77, 81925 Munich, Germany; 7 Department of Internal Medicine II, Klinikum am Bruderwald, Sozialstiftung Bamberg, Buger Straße 80, 96049 Bamberg, Germany; 8 Clinic of Orthopaedics and Sports Orthopaedics, School of Medicine, Technical University of Munich, Ismaninger Str. 22, 81675 Munich, Germany; 9 Program in Microbiology, Yale School of Medicine, New Haven, CT 06520, USA; 10 German Centre for Infection Research (DZIF), Partner Site Munich, Ismaninger Str. 22, 81675 Munich, Germany

**Keywords:** ascites, liver cirrhosis, SBP, secondary peritonitis, spontaneous bacterial peritonitis

## Abstract

Ascitic fluid infection is a serious complication of liver cirrhosis. The distinction between the more common spontaneous bacterial peritonitis (SBP) and the less common secondary peritonitis in patients with liver cirrhosis is crucial due to the varying treatment approaches. This retrospective multicentre study was conducted in three German hospitals and analysed 532 SBP episodes and 37 secondary peritonitis episodes. Overall, >30 clinical, microbiological, and laboratory parameters were evaluated to identify key differentiation criteria. Microbiological characteristics in ascites followed by severity of illness and clinicopathological parameters in ascites were the most important predictors identified by a random forest model to distinguish between SBP and secondary peritonitis. To establish a point-score model, a least absolute shrinkage and selection operator (LASSO) regression model selected the ten most promising discriminatory features. By aiming at a sensitivity of 95% either to rule out or rule in SBP episodes, two cut-off scores were defined, dividing patients with infected ascites into a low-risk (score ≥ 45) and high-risk group (score < 25) for secondary peritonitis. Overall, the discrimination of secondary peritonitis from SBP remains challenging. Our univariable analyses, random forest model, and LASSO point score may help clinicians with the crucial differentiation between SBP and secondary peritonitis.

## 1. Introduction

Spontaneous bacterial peritonitis (SBP) is a common life-threatening complication in patients with liver cirrhosis [1,2,3]. Less common in patients with liver cirrhosis is secondary peritonitis (SecP), which is triggered by an abdominal focus of infection such as intestinal perforation, abscess, or previous intra-abdominal surgery [3,4,5]. Due to their similar clinical presentations and different treatment approaches, the distinction between SBP and SecP is vital. To reduce the high mortality of SecP in patients with liver cirrhosis (50–80%) [3], clarification by abdominal computed tomography (CT) scan and, if necessary, prompt surgical intervention is mandatory [2,3]. By contrast, SBP is treated conservatively with antibiotics [1,2,3], as it is caused by translocation of bacteria [6]. Differentiation of SecP and SBP by computed tomography still enhances the risk of nephrotoxicity by intravenous contrast media, particularly for patients with liver cirrhosis and renal impairment [7,8,9,10].

Due to the rarity of SecP, with a prevalence of approximately 4.5% of all peritonitis cases in patients with liver cirrhosis [3,4], only a few studies have been published to date, including a maximum of less than 25 patients with liver cirrhosis suffering from SecP [3,11,12,13,14]. Previous attempts to assist clinicians with the specific but crucial need to distinguish SecP from SBP are even rarer. Runyon et al. established a score 30 years ago to distinguish SecP from SBP without the involvement of computed tomography analysing 15 SecP episodes [12,15]. The three ascitic fluid values of this point-score model, total protein > 1 g/dL, glucose < 50 mg/dL, and lactate dehydrogenase (LDH) above the upper limit of the normal value for serum, achieved a sensitivity and specificity of approximately 67% and 90%, respectively [3,12], and did not allow reliable prediction of SecP in a more recent study [14].

Given the critical importance of the distinction between SBP and SecP and the fact that the current gold standard of differentiation using CT imaging with contrast media poses a risk to patients with renal dysfunction, which is quite common in patients with liver cirrhosis suffering from infected ascites, we sought to help clinicians determine whether CT imaging is necessary to differentiate SBP from SecP. Thus, the purpose of this study is to provide clinicians with a tool to distinguish SecP from SBP based on >30 clinical, microbiological, and laboratory parameters. We performed a multicentre retrospective analysis including (1) a univariable comparison, (2) a random forest model ranking the importance of influencing factors, and (3) a LASSO model providing a point score.

## 2. Materials and Methods

### 2.1. Study Population

The study included patients with hydropic decompensated liver cirrhosis and infected ascites treated at three hospitals (University Hospital rechts der Isar of the Technical University of Munich, München Klinik GmbH Bogenhausen, RoMed Klinikum Rosenheim). Initially, all patients who underwent microbiological analysis of organ puncture were retrospectively identified using a microbiological database (HyBASE, Cymed, Bochum, Germany). In total, 9207 patients with microbiologic analysis of organ punctures were consecutively screened for study inclusion/exclusion.

Inclusion criteria: Episodes fulfilling all the following criteria were included: (1) age ≥ 18 years; (2) liver cirrhosis, and (3) infected ascites defined by ≥250 polymorphonuclear leucocytes/mm^3^ and/or leucocyte count ≥ 500/mm^3^.

Exclusion criteria: Episodes with infected ascites were excluded in the following cases: haemorrhagic, malignant, chylous, tuberculous, or pancreatic ascites; continuous ambulatory peritoneal dialysis peritonitis; and insufficient documentation.

**Figure 1:**
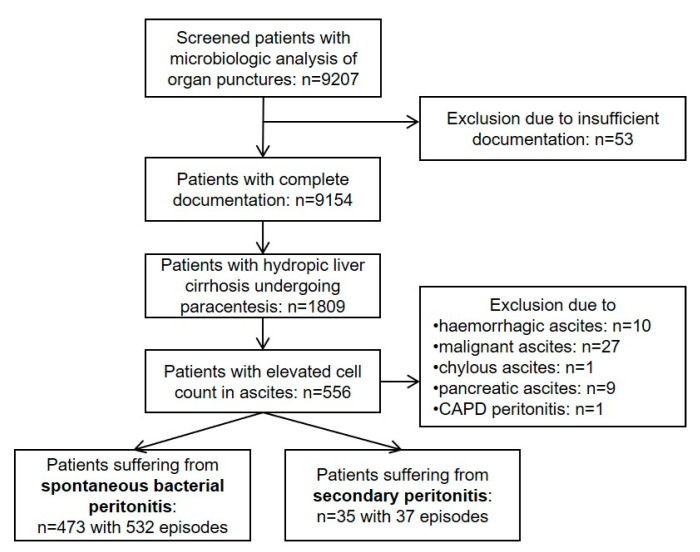
Flow chart of the screening and eligibility process of the study. CAPD, continuous ambulatory peritoneal dialysis.

Based on these inclusion and exclusion criteria, 532 SBP episodes (473 patients) and 37 SecP episodes (35 patients) were identified. Figure 1 illustrates the detailed information on the screening and eligibility processes of the study.

### 2.2. Definition of SecP

SecP is defined as a peritoneal infection secondary to intra-abdominal lesions such as spontaneous perforation of the hollow viscus (*n* = 23; perforation of the upper gastrointestinal tract, *n* = 8; perforation of the lower intestinal tract, *n* = 15) or penetrating infectious/necrotic processes (*n* = 9; appendicitis, *n* = 2; cholecystitis, *n* = 1; liver abscess, *n* = 1; necrotic bowel due to ischemia/incarceration, *n* = 5). SecP following intra-abdominal surgery (*n* = 5) included hemicolectomy for colorectal cancer resection (*n* = 2), emergency laparotomy due to unstoppable haemorrhage (*n* = 2), and repair of abdominal wound dehiscence (*n* = 1). Two patients experienced more than one episode, with each perforation occurring at different sites in the lower or upper intestinal tract.

### 2.3. Assessment of Predictors

The severity of liver cirrhosis was assessed using the Child–Pugh score, the model for end-stage liver disease with serum sodium (MELD-Na) score, and the acute-on-chronic liver failure (ACLF) score [1,2,16,17,18]. Due to missing data, the ACLF score was slightly modified according to CLIF-C ACLF criteria [19] as follows: serum creatinine ≥ 2 mg/dL; bilirubin > 12 mg/dL; INR ≥ 2.5; encephalopathy; and therapy with vasopressors or mechanic ventilation (intubation). The Charlson Comorbidity Index, which predicts the 10-year mortality risk in patients suffering from comorbidities, was adjusted to age [20,21,22]. Regarding the isolation of pathogens, coagulase-negative staphylococci were considered as contamination and excluded from the analyses.

### 2.4. Ethics

This study was approved by the Ethics Committee at the Technical University of Munich, School of Medicine, University Hospital rechts der Isar, Munich, Germany, with approval no. 201/19 S-SR, and conducted in accordance with the Declaration of Helsinki. The institutional review board waived the requirement for written consent because of the retrospective study design (World Health Organization trial registration number: DRKS00017728).

### 2.5. Sample Method and Microbiological Analysis

Due to the retrospective study design, the collection of microbiological cultures was not standardised. In general, 10–20 mL of ascitic fluid and optionally 10–20 mL of blood were collected, inoculated into one aerobic and one anaerobic blood culture bottle (BacTec system, Becton Dickinson, Heidelberg, Germany), and incubated at 37 °C for 5 days. Microbial identification was performed using biochemical testing systems (ATB, API, VITEK system, BioMérieux, Nurtingen, Germany) or matrix-associated laser desorption/ionization-time of flight (MALDI-TOF, Bruker Corporation, Billerica, MA, USA).

### 2.6. Statistical Methods

Preparation for statistical analyses: variables missing >30% of the values (albumin, alkaline phosphate, carcinoembryonic antigen, glucose, lactate, LDH, procalcitonin, and pH, as well as respective serum:ascites ratios) were excluded from the analyses. Missing values of other variables were imputed using the random forest-based ‘MissForest’ algorithm [23]. Information on group membership in SBP and SecP was excluded from this imputation to prevent associations being generated between this outcome and the predictor variables. Episodes were counted as being different if the patients were discharged from the hospital for at least two weeks without relapse.

Univariable statistical analyses: The distribution of quantitative and qualitative data is presented as the median (range) or absolute and relative frequencies, respectively. Group comparisons were performed using Fisher’s exact test or Pearson’s Chi-squared test on qualitative variables and Wilcoxon’s rank-sum test for quantitative variables.

Random forest model: the rank order of importance of predictors for diagnosis was evaluated by a conditional inference random forest model using the internally validated out-of-bag permutation importance measure [24,25]. A random forest model was chosen for this purpose to cover potentially interacting and non-linear effects.

Least absolute shrinkage and selection operator (LASSO) regression model: the LASSO regression model was used to obtain an additive score model for dichotomous predictors. Dichotomization was performed through predefined and established cut-off values (ACLF score > 2, Child–Pugh score > 9) or through the computation of optimal cut-off values determined by maximised statistics [26]. Shrinkage of the parameters of the LASSO model was performed using the maximum value of the area under the curve (AUC) according to the “1se” rule and using five-fold cross-validation. Subsequently, the resulting model coefficients were divided by the smallest coefficient and rounded to the nearest integer to yield an additive point-score model. The prognostic accuracy of the point-score model and of ‘optimal’ cut-off values that achieve a sensitivity and a specificity of 95% in learning data was internally validated by five-fold cross-validation. The relative frequencies of SBP and SecP were maintained and the dichotomization of variables, the building of the point-score model, and the determination of the ‘optimal’ cut-off values were repeated in the folds of the cross-validation to obtain unbiased performance estimates. Fagan’s nomogram (or Bayes theorem) describes the post-test probability following the LASSO scoring model to detect SecP.

Statistical hypothesis testing was performed using two-sided exploratory significance levels of 0.05. All statistical analyses and the web application were performed using R version 4.0.3 (R Foundation for Statistical Computing, Vienna, Austria).

## 3. Results

### 3.1. Baseline Characteristics

Overall, 532 SBP episodes (473 patients) and 37 SecP episodes (35 patients) were analysed in this retrospective multicentre study. The median length of hospital stay was 27 days (range 1–287) and the overall mortality rate was 40.2% (204/508). The most frequently isolated pathogens were Enterobacteriaceae (26.7%, 152/569), followed by *Enterococcus* spp. (12.3%, 70/569), *Candida* spp. (7.6%, 43/569), anaerobes (2.6%, 15/569), and *Pseudomonas* spp. (1.2%, 7/569). In total, 314 of 532 SBP episodes and 31 of 37 SecP episodes were non-community-acquired (*p* = 0.005).

### 3.2. Univariable Analyses

Table 1 and the Supplementary Table S1 illustrate univariable analyses comparing >30 clinical, laboratory, and microbiological features between patients with SBP and SecP. Patients with SecP were admitted to the intensive care unit (ICU) more frequently and stayed longer in the hospital than patients with SBP (32 versus 17 days in median, *p* = 0.005). In-hospital mortality was higher in patients with SecP than with SBP (45.7% versus 39.7%). Patients with SecP had higher median inflammatory markers (C-reactive protein: 10.4 vs. 6.1 mg/dL, leucocytes: 12.7 vs. 9.1 G/L). The median leucocyte count in ascites of patients with SecP and SBP was 4.0 vs. 1.5 G/L, respectively (*p* = 0.027). Pathogen isolation was successful in 78.4% of the SecP group versus 42.1% of the SBP group. *Candida* spp., *Enterococcus* spp., and anaerobic bacteria were significantly more prevalent in pathogen-positive SecP episodes than in pathogen-positive SBP episodes (44.8% vs. 13.4%, *p* < 0.001; 72.4.8% vs. 21.9%, *p* < 0.001; and 17.2% vs. 4.5%, *p* = 0.002, respectively). Polymicrobial ascites in pathogen positive episodes was observed more often in the SecP group than in the SBP group (62.1% vs. 16.5% of all episodes, *p* < 0.001). Pathogen persistence in ascites >3 days in pathogen positive episodes was observed more frequently in patients with SecP than in those with SBP (34.5% versus 13.4%, *p* < 0.001).

Two independent statistical approaches, random forest and LASSO regression, were used to identify the key distinguishing features between SecP and SBP and to produce a diagnostic score, respectively.

### 3.3. Random Forest Model: Important Features

**Figure 2:**
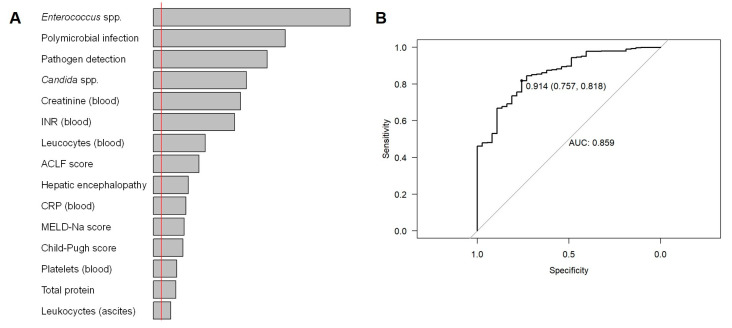
Random forest analysis. (**A**) Most important features based on random forest analysis (red line: absolute value of the lowest observed negative importance measure). (**B**) Receiver operating curve (ROC) and area under the curve (AUC) = 0.859. ACLF, acute-on-chronic liver failure; CRP, C-reactive protein; INR, international normalised ratio; MELD-Na, model for end-stage liver disease with serum sodium.

Figure 2 displays the most important features for differentiation between SecP and SBP as determined by the random forest model. Pathogen detection, particularly for *Enterococcus* spp., *Candida* spp., and polymicrobial ascites, was most indicative, followed by creatinine level, international normalised ratio (INR), and leucocytes in the blood. Internal validation of the random forest model revealed an area under the receiver operating characteristic curve (out-of-bag AUC) of 0.86.

### 3.4. LASSO Regression Point-Score Model

**Figure 3:**
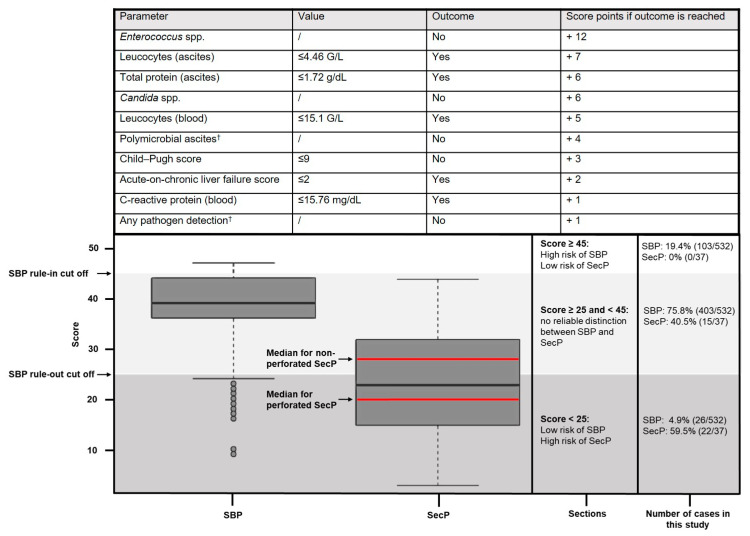
Point score model including a rule-in cut off (score ≥ 45) and a rule-out cut off (score < 25) for SBP, dividing the study patients into a low-risk and high-risk group for SecP. † Except coagulase negative *Staphylococcus* spp.; median scores for the subgroups of SecP (perforated and non-perforated SecP episodes) illustrated in red.

Based on the LASSO regression model, an additive point score was established, proposing ten decision criteria for the differentiation of SecP from SBP (Figure 3). Counting from zero to 47, the probability of SBP increases and that of SecP lowers as the model predicts SBP.

**Figure 4:**
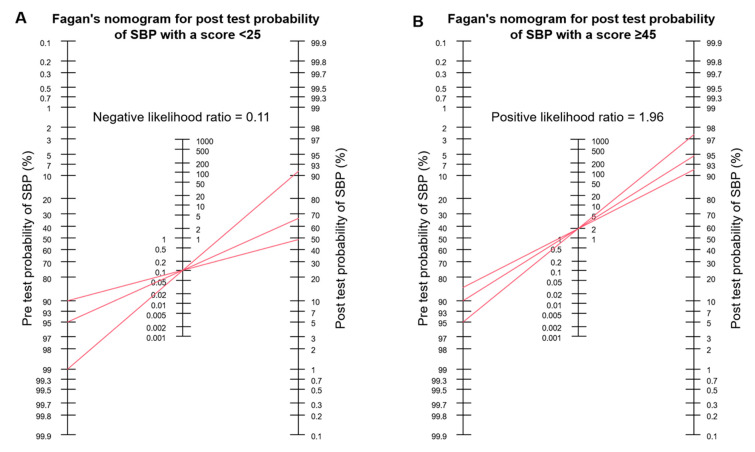
Fagan’s nomograms for post-test probability. (**A**) Post-test probability of SBP for a point-score model < 25. Referring to a pre-test probability of 95%, 90%, and 85% for SBP (corresponding to a prevalence of 5%, 10%, and 15% for SecP), of all cases with infected ascites, a score < 25 leads to a post-test probability of 66.7%, 48.6%, and 37.4%, respectively. (**B**) Post-test probability of SBP for a point-score model ≥ 45. Assuming a pre-test probability for SBP of 95%, 90%, and 85% by referring to all cases with infected ascites (corresponding to a prevalence of 5%, 10%, and 15% for SecP), a score ≥45 results in a positive predictive value of 97.4%, 94.6%, and 91.7%, respectively.

Cut offs for the rule in and the rule out of SBP episodes were defined as follows, dividing patients with infected ascites into a low-risk (score ≥ 45) and high-risk group for SecP (score < 25):

Cut off for the rule out of SBP episodes: by aiming at a sensitivity ≥ 95%, the ideal cut off for the model to rule out SBP episodes was determined at a score < 25, meaning that at least 95% of cases with SBP had a score ≥ 25. The median cross-validated sensitivity was 95.7%, implying that 95.7% of SBP episodes are expected to have a score ≥ 25, and 4.3% of SBP episodes are expected to have a score < 25 in external data. The median cross-validated specificity was 41.0%, illustrating that 41.0% of SecP are expected to reach a score < 25, and 59% are expected to reach a score ≥ 25. Fagan’s nomograms (Figure 4A) were used to assess the post-test probability of SBP. Referring to a pre-test probability of 95%, 90%, and 85% for SBP (corresponding to a prevalence of 5%, 10%, and 15% for SecP), of all cases with infected ascites, a score < 25 leads to a post-test probability of 66.7%, 48.6%, and 37.4%, respectively.

Cut off for the rule in of SBP episodes: by aiming at a specificity ≥ 95%, the ideal cut-off score for the rule in of SBP episodes was determined at a score ≥ 45. The median cross-validated specificity was 92.1%, meaning that 92.1% of SecP episodes are expected to have a score < 45 in external data. Assuming a pre-test probability for SBP of 95%, 90%, and 85% by referring to all cases with infected ascites (corresponding to a prevalence of 5%, 10%, and 15% for SecP), a score ≥ 45 results in a positive predictive value of 97.4%, 94.6%, and 91.7%, respectively (Figure 4B).

As illustrated in Figure 3, 73.5% of episodes with infected ascites (75.8% SBP episodes, 40.5% SecP episodes) were assigned to the group in between the low- and high-risk groups for SBP, allowing no reliable distinction between SBP and SecP. A total of 59.5% (22/37) of SecP episodes were assigned to the high-risk group for SecP, and no SecP episode fell into the low-risk group. The median scores of SBP and SecP episodes were 39 and 23, respectively. Concerning SecP subgroups, the median score of non-perforated SecP episodes (*n* = 14) was 28, whereas the median score of perforated SecP episodes (*n* = 23) was 20.

## 4. Discussion

SecP represents a serious disease in patients with liver cirrhosis, and its prompt differentiation from SBP is paramount [1,2]. Management of the two entities is divergent, with SBP being treated conservatively and SecP often requiring surgery [1,2,3,5]. However, distinguishing SBP from SecP is challenging in clinical practice because both patient groups exhibit similar symptoms, such as fever and abdominal pain [27,28].

In our study, the incidence of SecP in patients with infected ascites among all patients with SecP and SBP was low at 6.9% (35/508 patients), which is comparable to the previously reported prevalence of 4.5% of this rare disease [3,4].

In univariable analysis, >30 clinical, laboratory, and microbiological parameters as well as four clinical scores were compared between the SecP (37 episodes) and the SBP group (532 episodes). Higher C-reactive protein levels, leucocyte counts, and creatinine levels in serum were significantly predicting SecP. Severity of illness as evidenced by organ failure (renal failure, hepatic failure/INR elevation, or encephalopathy), and systemic inflammatory markers (leucocytes, C-reactive protein) were also identified as significant differentiators between SBP and SecP in the random forest model and the LASSO point score. Patients with SecP were more severely ill, had a higher rate of organ failure and admission to the ICU, and longer hospital stay than patients with SBP. Consistent with our findings, Soriano et al. have revealed in a retrospective analysis that patients with SecP had a significantly higher inflammatory response than patients with SBP [3]. Apart from morbidity, mortality was found to be high for SBP (39.7%) and SecP (45.7%), respectively, with a substantial but non-significant percentage difference. In contrast, Soriano et al. found a significant difference in mortality between SBP (26.4%) and SecP (66.6%) [3], whereas Ruault et al. identified a non-significant difference between SBP- (81.0%) and SecP- (77.5%) 1-year mortality in their ICU cohort [14]. The major reason for these inconsistent study findings may be due to different times of diagnosis and treatment approaches in patients with SecP. Early time of diagnosis and prompt surgical intervention is crucial for survival in patients with liver cirrhosis suffering from SecP. Furthermore, severity of illness and the underlying comorbidities at time of diagnosis also play an important role for survival in the case of SecP or SBP. Leucocyte cell count in ascites was significantly higher in the SecP group (median 4.0 G/L) compared to the SBP group (median 1.5 G/L). A cut-off level of 4.46 G/L was determined by LASSO regression for the leucocyte count in ascites to best distinguish between the two groups. This marker also proved to be a good differentiator in other studies [11,12,14]. Aside, previous length of stay in the hospital before diagnosis was significantly higher in SecP patients compared to the SBP group. Regarding clinical scores, an ACLF score > 1 provided differentiation between SecP and SBP in univariable analysis. Interestingly, patients with a high Child–Pugh score were more likely to have SBP than those with SecP. This might be related to the higher risk of developing SBP in patients with progressive liver cirrhosis [29].

Both the random forest and LASSO model weighted microbiological features such as the presence of *Enterococcus* spp., *Candida* spp., polymicrobial ascites, or the isolation of any pathogen in ascites as the most important criteria for distinguishing SBP from SecP. Additionally, the univariable analysis revealed that patients with SecP had significantly more anaerobes, and pathogen persistence in ascites was longer than SBP. In contrast to SecP, anaerobes play a minor role in SBP, as previously reported [3,30,31,32,33]. Therefore, current guidelines consider antibiotics such as third-generation cephalosporins with low coverage against anaerobes to be sufficient empiric treatment for community-acquired SBP [2,28,33,34]. Sheckman et al. hypothesised that the low isolation rate of anaerobes in SBP may be attributed to the relatively high partial pressure of oxygen in ascitic fluid, restricting the growth of anaerobes [35]. In line with our findings, Jang et al. analysed the pathogen spectrum of 419 patients undergoing emergency surgery for intestinal perforation and identified *Enterococcus* spp. as the predominant pathogen, followed by Enterobacteriaceae and *Candida* spp. [36]. Thus, polymicrobial ascites and the isolation of *Candida* spp. or *Enterococcus* spp. are strong indicators of the presence of SecP [4]. A drawback of microbiological discrimination features is the time to positivity of microbiological diagnostic methods. Microbiological parameters account for four of the ten decisive scoring indicators identified by the LASSO model. This high relevance of microbiological diagnostics delays the scoring result, impairing the fast-decision-making process, if ascites analysis has not yet been performed. Although some microbiological results are indicative in the first hours due to rapidly growing organisms and/or sophisticated diagnostics, we ensured that the score can be entered without microbiological test results and that the score increases from 0 when predicting SBP. A user-friendly web application is provided online [37].

Previous attempts to assist clinicians in distinguishing SecP from SBP are rare. In particular, Runyon’s criteria are discussed in the literature, assuming SecP when two of the three following criteria are met in ascitic fluid: glucose level < 50 mg/dL, protein concentration > 10 g/L, or LDH above the upper limit of the normal value for serum [12]. However, Runyon’s criteria have been described with an insufficient sensitivity of 67% and low positive predictive value in view of the low prevalence of SecP [3,14]. A more recent approach, which focused specifically on patients managed in the ICU, was performed by Ruault et al. and included 21 patients with SecP [14]. The main differentiation factors between SecP and SBP were ascitic leucocyte count > 10 G/L and absence of laboratory signs of decompensated cirrhosis defined as platelet count < 150 G/L, and/or bilirubin > 2.9 mg/dL, and/or prothrombin time < 40% [14]. The significantly higher leucocyte count in ascites in SecP is confirmed in our study and is consistent with the pathophysiological understanding of SecP, originating from intra-abdominal lesions. This retrospective ICU study also validated Runyon criteria, which were only assessed in <50% of study patients and did not allow independent prediction of SecP [14].

Like previous studies [12,14], we faced the problem of finding a cut off in our statistical approach with high predictive values for SecP for this rare but lethal disease. Thus, we decided to determine two cut offs for SBP episodes, either to rule in or rule out SBP episodes. Since the discrimination accuracy of our model depends on a considerable extent on the prevalence of SecP and SBP, Fagan’s nomogram was selected to best illustrate the negative and positive predictive values. The cut off for the rule in of SBP episodes was determined at a score ≥ 45. Given a prevalence of 95% for SBP (corresponding a prevalence of 5% for SecP) in patients with infected ascites, the positive test result (score ≥ 45) with respect to SBP was associated with a post-test probability of 97.5%. The cut off for the rule out of SBP was set at a score < 25. Based on the same prevalence of 95% and 90% for SBP, a score < 25 is associated with a negative predictive value of 33.3% and 51.4% for SBP, corresponding to the post-test probabilities for SecP. Based on these two cut-off values, patients with infected ascites were divided into a low-risk and high-risk group for SecP, shown in Figure 3.

The main novelty of our study is the new possibility of good discrimination of SBP and SecP of patients belonging to the low- or high-risk group for SecP (score ≥ 45 or <25, 26.5% of episodes). SecP can be ruled out in patients with a score ≥ 45. In contrast, SecP is highly probable in patients with a score < 25 and therefore should undergo CT-scan to rule out SecP. In addition, ranking the importance of discriminatory parameters by the random forest model is helpful for clinicians in the decision-making process.

Limitations of our study particularly include the retrospective design owing to the rarity of SecP, which also explains the challenge to comprehensively collect clinical, microbiological, and laboratory parameters in the present study. Thus, promising parameters for distinguishing SBP and SecP such as carcinoembryonic antigen (CEA) could not be included to the analysis [13]. The major disadvantage of the LASSO score model is that most episodes (73.5%, *n* = 418/569) with infected ascites were not assigned to the low- or high-risk group for SecP, allowing no clear distinction between SBP and SecP for these patients.

In conclusion, despite the difficulties reported by us and previously in the literature to generate a highly sensitive and specific score [3,12,14], the LASSO model of this study is a tool for clinicians helping to identify patients suffering from cirrhosis and infected ascites with a high risk for SecP (score < 25) who should undergo an abdominal CT scan, as well as patients with a low risk for SecP (score ≥ 45) who might not profit from CT imaging. Moreover, the univariable and random forest analyses could further help in the difficult distinction between SecP and SBP in routine clinical practice. However, distinguishing SecP from SBP in patients with liver cirrhosis remains challenging. The addition of promising parameters such as CEA to our LASSO model could help to increase its discriminatory power in further prospective studies. Further multicentre studies with a high number of episodes and sophisticated statistical approaches are needed.

## Figures and Tables

**Table 1:** Comparison of SecP and SBP episodes. Parameters are displayed as relative frequency in % (absolute frequency) or median (range). Parameters marked with † were not considered for the random forest and LASSO regression model. All parameters are based on episodes apart from sex and mortality, which are based on patients. The blood parameter creatinine was adapted to dialysis as previously described for MELD score calculations. ICU, intensive care unit; SecP, secondary peritonitis; SBP, spontaneous bacterial peritonitis; NA, not available.

Parameters	SecP Episodes (*n* = 37; 35 Patients)	SBP Episodes (*n* = 532; 473 Patients)	*p*-Value
Clinical parameters			
Age (years) ^†^	63 (45–92)	63 (23–88)	0.573
Female patients ^†^	20.0% (7/35)	25.2% (119/473)	0.911
Length of stay (days) ^†^	32 (2–124)	17 (1–287)	0.005
ICU admission ^†^	73.0% (27/37)	44.5% (237/532)	0.001
Mortality ^†^	45.7% (16/35)	39.7% (188/473)	0.428
Laboratory parameters			
Creatinine in serum (mg/dL)	2.9 (1.0–4.0; 1 NA)	1.7 (0.9–4.0; 9 NA)	0.01
C-reactive protein in serum (mg/dL)	10.4 (1.1–34.3; 5 NA)	6.1 (0.1–32.7; 84 NA)	0.004
Leucocytes in blood (G/L)	12.7 (3.2–27.8)	9.1 (1.2–41.0; 6 NA)	0.007
Leucocytes in ascites (G/L)	4.0 (0.5–70.3)	1.5 (0.1–146.0)	0.027
Microbiologic parameters			
Pathogen detection	78.4% (29/37)	42.1% (224/532)	<0.001
Polymicrobial infection	62.1% (18/29)	16.5% (37/224)	<0.001

## Data Availability

The raw data are not publicly available as they contain extensive information that could compromise the privacy of research participants, but they are available from the corresponding author upon reasonable request.

## Supplementary Materials

The following Supporting Information can be downloaded at: https://www.mdpi.com/article/10.3390/diagnostics13050994/s1, Supplementary Table S1: Comparison of SecP and SBP episodes.

## Abbreviations

ACLF, acute-on-chronic liver failure; AUC, area under the curve; CRP, C-reactive protein; CT, computed tomography; ICU, intensive care unit; INR, international normalised ratio; LDH, lactate dehydrogenase; MELD, model for end-stage liver disease; NA, non-available; SBP, spontaneous bacterial peritonitis; SecP, secondary peritonitis.
