# Supplementary material for: Differentiation of Spontaneous Bacterial Peritonitis from Secondary Peritonitis in Patients with Liver Cirrhosis: Retrospective Multicentre Study
Source: Diagnostics (Basel). 2023 Mar 6;13(5):994. doi: 10.3390/diagnostics13050994 (PMC10000989; doi:10.3390/diagnostics13050994)
Supplement: Supplementary file 1 [file diagnostics-13-00994-s001.zip › diagnostics-2195552-supplementary.pdf]

Differentiation of spontaneous bacterial peritonitis from secondary peritonitis in patients with liver cirrhosis: Retrospective multicentre study

Supplementary Material

Supplementary Table: Comparison of SecP and SBP episodes (full table). Parameters are displayed as relative frequency in % (absolute frequency) or median (range). Parameters marked with † were not considered for the random forest and LASSO regression model. All parameters are based on episodes apart from sex and mortality, which is based on patients. The blood parameter creatinine was adapted to dialysis as previously described for MELD score calculations.[27] SecP, secondary peritonitis; SBP, spontaneous bacterial peritonitis; NA, not available; MELD-Na, model for end-stage liver disease with serum sodium.

**Parameters**	**SecP episodes (n=37; 35 patients)**	**SBP episodes (n=532; 473 patients)**	**P-value**
**Clinical parameters**			
Age (years)†	63 (45–92)	63 (23–88)	0.573
Female patients†	20.0% (7/35)	25.2% (119/473)	0.911
Previous hydropic decompensation of liver cirrhosis	82.1% (23/28; 9 NA)	74.9% (341/455; 77 NA)	0.527
Previous SBP episodes <3 months	8.1% (3/37)	17.4% (91/524; 8 NA)	0.165
Previous upper gastrointestinal bleeding <3 months	5.4% (2/37)	6.4% (33/517; 15 NA)	1.000
Previous hepatic encephalopathy	0% (0/37)	5.1% (27/532)	0.246
Cancer disease	16.2% (6/37)	22.3% (118/529; 3 NA)	0.509
Diabetes	35.1% (13/37)	32.5% (173/532)	0.883
Chronic renal failure	24.3% (9/37)	22.0% (117/531; 1 NA)	0.905
Previous kidney or liver transplant	2.7% (1/37)	2.6% (14/532)	1
Immunosuppressive therapy	22.9% (8/35; 2 NA)	13.5% (70/517; 15 NA)	0.133
Length of stay (days)†	32 (2–124)	17 (1–287)	0.005
Number of ascitic punctures†	2 (1–17)	3 (1–22)	0.554
ICU admission†	73.0% (27/37)	44.5% (237/532)	0.001
First diagnosis on ICU	29.7% (11/37)	17.0% (90/530; 2 NA)	0.189
Pleural effusion	43.2% (16/37)	40.7% (210/516; 16 NA)	0.755
Oesophageal varices	60.0% (21/35; 2 NA)	63.0% (308/489; 43 NA)	0.865
Acute upper gastrointestinal bleeding	5.4% (2/37)	12.6% (67/532)	0.296
Hepatic encephalopathy	29.7% (11/37)	37.6% (200/532)	0.434
Mortality†	45.7% (16/35)	39.7% (188/473)	0.428
**Laboratory parameters**			
Creatinine in serum (mg/dL)	2.9 (1.0–4.0; 1 NA)	1.7 (0.9–4.0; 9 NA)	0.010
C-reactive protein in serum (mg/dL)	10.4 (1.1–34.3; 5 NA)	6.1 (0.1–32.7; 84 NA)	0.004

Bilirubin in serum (mg/dL)	3.4 (0.2–31.2; 1 NA)	3.6 (0.2–46.4; 78 NA)	0.239
Glucose in serum (mg/dL)	124 (9–330; 11 NA)	118 (18–408; 140 NA)	0.955
Leucocytes in blood (G/L)	12.7 (3.2–27.8)	9.1 (1.2–41.0; 6 NA)	0.007
Platelets in blood (G/L)	160 (15–676; 1 NA)	112 (4–674; 5 NA)	0.080
Internationalized normalized ratio	1.4 (1.0–3.8)	1.5 (0.9–7.1; 47 NA)	0.247
Leucocytes in ascites (G/L)	4.0 (0.5–70.3)	1.5 (0.1–146.0)	0.027
Total protein in ascites (g/dL)	1.94 (0.29–3.74, 11 NA)	1.40 (0.19–5.82, 110 NA)	0.419
**Microbiologic parameters**			
Pathogen detection	78.4% (29/37)	42.1% (224/532)	<0.001
Candida spp.	44.8% (13/29)	13.4% (30/224)	<0.001
Enterococcus spp.	72.4% (21/29)	21.9% (49/224)	<0.001
Anaerobe bacteria	17.2% (5/29)	4.5% (10/224)	0.002
Persistent detection of pathogens in ascites three days after diagnosis	34.5% (10/29)	13.4% (30/224)	<0.001
Polymicrobial infection	62.1% (18/29)	16.5% (37/224)	<0.001
**Clinical scores**			
Acute-on-chronic liver failure score >1	71.4% (25/35, 2 NA)	53.6% (231/431, 101 NA)	0.007
Child–Pugh score	10 (7–14, 1 NA)	10 (7–15, 98 NA)	0.697
MELD-Na score	26.9 (11.7–40.0; 2 NA)	26.3 (6.2–40.0; 108 NA)	0.850
Charlson Comorbidity Index	7 (4–14)	7 (3–20)	0.840
